# Considering Motor Excitability During Action Preparation in Gambling Disorder: A Transcranial Magnetic Stimulation Study

**DOI:** 10.3389/fpsyt.2020.00639

**Published:** 2020-06-30

**Authors:** Caroline Quoilin, Julien Grandjean, Julie Duque

**Affiliations:** CoActions Lab, Institute of Neuroscience, Université catholique de Louvain, Brussels, Belgium

**Keywords:** gambling disorder, addiction, inhibitory control, impulsivity, transcranial magnetic stimulation, motor system, action preparation

## Abstract

A lack of inhibitory control appears to contribute to the development and maintenance of addictive disorders. Among the mechanisms thought to assist inhibitory control, an increasing focus has been drawn on the so-called preparatory suppression, which refers to the drastic suppression observed in the motor system during action preparation. Interestingly, deficient preparatory suppression has been reported in alcohol use disorders. However, it is currently unknown whether this deficit also concerns behavioral, substance-free, addictions, and thus whether it might represent a vulnerability factor common to both substance and behavioral addictive disorders. To address this question, neural measures of preparatory suppression were obtained in gambling disorder patients (GDPs) and matched healthy control subjects. To do so, single-pulse transcranial magnetic stimulation was applied over the left and the right motor cortex to elicit motor-evoked potentials (MEPs) in both hands when participants were performing a choice reaction time task. In addition, choice and rapid response impulsivity were evaluated in all participants, using self-report measures and neuropsychological tasks. Consistent with a large body of literature, the MEP data revealed that the activity of the motor system was drastically reduced during action preparation in healthy subjects. Surprisingly, though, a similar MEP suppression was observed in GDPs, indicating that those subjects do not globally suffer from a deficit in preparatory suppression. By contrast, choice impulsivity was higher in GDPs than healthy subjects, and a higher rapid response impulsivity was found in the more severe forms of GD. Altogether, those results demonstrated that although some aspects of inhibitory control are impaired in GDPs, these alterations do not seem to concern preparatory suppression. Yet, the profile of individuals suffering of a GD is very heterogeneous, with only part of them presenting an impulsive disposition, such as in patients with alcohol use disorders. Hence, a lack of preparatory suppression may be only shared by this sub-type of addicts, an interesting issue for future investigation.

## Introduction

Self-regulation is essential to behave in a goal-directed manner. In particular, the ability to suppress prepotent but inappropriate responses is a key component, preventing one to respond to stimulus-driven impulses (1, 2). Without the efficient operation of this inhibitory control, behavior becomes maladaptive, as evidenced in a range of psychiatric disorders, including addictive disorders (3, 4). As such, a core element of addiction is a loss of control over either the use of a substance or the engagement in a recurrent activity, despite awareness of negative consequences, which clearly interfere with long-term goals.

Among the different processes assisting inhibitory control, an increasing focus has been drawn on mechanisms allowing to downregulate the excitability of the motor system (5). Accordingly, a drastic suppression of motor activity has been reported when subjects are in the process of stopping an action (6, 7), but also during the preparation of motor acts (8, 9). In particular, by measuring motor-evoked potentials (MEPs) elicited by single-pulse transcranial magnetic stimulation (TMS) over the primary motor cortex (M1), studies have monitored changes in the excitability of the corticospinal pathway during instructed-delay choice reaction time (RT) tasks (10–14). Such tasks typically require participants to choose between responding with the left of the right hand according to an informative preparatory cue, and to withhold their response until the onset of an imperative signal. When TMS pulses are applied between the cue and the imperative, the amplitude of MEPs probed in both hands are strongly reduced relative to resting conditions (15–17). This phenomenon, referred to as preparatory suppression (or inhibition), is thought to help prevent premature or inappropriate motor responses and, more generally, to ensure some sort of impulse control (8, 18–20).

Consistent with this view, preparatory suppression appears to be deficient in individuals lacking inhibitory control, such as in addictive disorders. We have recently shown that alcohol-dependent patients (ADPs) display a reduced MEP suppression during action preparation relative to matched healthy participants (9), suggesting that a shortage of preparatory suppression might represent a newly identified feature of addictive disorders. Moreover, it might serve as an objective indicator of addiction severity, as the magnitude of this defect was linked to the subsequent propensity to relapse.

Chronic alcohol consumption has considerable neurotoxic effects, with the most pronounced damage reported in regions underpinning response inhibition, such as the frontal lobes and basal ganglia (21–23). In addition, the degree of brain atrophy is related to the amount of alcohol previously consumed (24). Hence, the deficit in preparatory suppression could be a consequence of brain damage induced by chronic alcohol exposure. Alternatively, a lack of inhibitory control might have been present before the pathology, predisposing individuals to early recreational experiences with alcohol, or facilitating their transition towards alcohol use disorder. For example, offspring of ADPs, known to be at higher risk of developing alcohol use disorders (25), display deficient inhibitory control (26, 27). In addition, impulsivity assessed during childhood or adolescence predicts substance use disorders later in life (28, 29).

Inhibitory control is also impaired in behavioral, substance-free, addictions, implying that it might act as a vulnerability factor common to both substance and behavioral addictive disorders (3, 30). This matter has been especially addressed in gambling disorder (GD), which shares considerable phenomenological parallels with substance addiction, including difficulties to control the urge to gamble despite awareness of its negative impact, unsuccessful attempts to cut back, or the emergence of craving in front of gambling-related cues (31). In particular, an increasing body of literature has highlighted that patients suffering from GD (GDPs) have higher levels of impulsivity and lower response inhibition abilities than control subjects (32–34). Moreover, several studies have reported an interesting association between those alterations and gambling severity (35–37).

The goal of the present study was to determine whether GDPs, who suffer from an addictive disorder but are preserved from the neurotoxic influence of drugs of abuse, display a lack of preparatory suppression in the motor system, similar to our findings in ADPs. To test this idea, we applied single-pulse TMS over the left and the right M1 to elicit MEPs in GDPs and matched healthy control subjects performing an instructed-delay choice RT task. The study also involved the examination of other aspects of inhibitory control, including trait impulsivity, choice impulsivity, and response inhibition. Based on the hypothesis that a lack of preparatory suppression represents a common feature to both substance and behavioral addictions, we expected preparatory suppression to be less pronounced in GDPs than in healthy subjects.

## Materials and Methods

### Participants

Thirteen right-handed individuals with a diagnosis of GD were included in the study. All patients were recruited through advertisements in several gambling areas, such as in casinos and sports betting facilities, and through a collaboration with the psychiatry unit of the Saint-Luc Academic Hospital (Université catholique de Louvain, Brussels, Belgium). Gambling dependence severity was assessed before the experiment using the South Oaks Gambling Scale (SOGS); a score higher than 5 was required to participate, indicating probable pathological gambling (38). Based on this criterion, we selected 16 participants. Moreover, on the day of the experiment, a face-to-face clinical interview was conducted by an experienced psychologist, and only patients who met DSM-5 criteria for GD (39) were kept in the final sample (n = 13). All patients gambled at least more than once a week; the mean duration of gambling behavior was 6.1 years (SD = 3.86). Their main gambling activity was either sports betting (n = 7) or online casino games (n = 6). GDPs were matched for age, gender, and education level with 13 right-handed healthy control subjects (HCs); all controls had a SOGS score of 0. Exclusion criteria for both groups included major neurological or psychiatric disorder, any drug treatment that could influence performance or neural activity (including benzodiazepine), and no history of substance use disorder (except nicotine). Nicotine dependence was more prevalent among GDPs (n = 4) than controls (n = 0). Finally, in order to avoid any confounding effects due to a problematic consumption of alcohol, subjects from both groups had to complete the Alcohol Use Disorder Test (AUDIT); a score higher than 10 was considered as an exclusion criterion (40). All participants gave written informed consent, following a protocol approved by the Biomedical Ethic Committee of the Saint-Luc University Hospital, Université catholique de Louvain. All the experimental procedures occurred at the Institute of Neuroscience of the Université catholique de Louvain, and a 50-euro voucher was provided at the end of the experiment as a financial compensation.

### Experimental Procedure

#### Self-Reported Measures

Current clinical status was measured using French versions of the Spielberger State Trait Anxiety Inventory [STAI Trait and State; (41, 42)] and the Beck Depression Inventory [BDI; (43, 44)]. To evaluate trait impulsivity, both the Barratt Impulsiveness Scale Version 11 [BIS-11; (45, 46)] and the UPPS Impulsive Behavior Scale (47, 48) were used. While the former is composed of three subscales, namely attentional, motor, and non-planning impulsivity, the latter allows to assess four different dimensions of impulsivity, referred to as urgency, lack of premeditation, lack of perseverance, and sensation seeking. Finally, choice impulsivity was measured using the Monetary Choice Questionnaire [MCQ; (49)]. This tool consists of 27 dichotomous choices between smaller-immediate and larger-delayed monetary rewards to provide individual's delay discounting rate, i.e. the *k-*value. Three magnitudes are assessed, resulting in separate discounting rates for small, medium, and large reward; an overall discounting rate was also obtained. *K*-values can range from 0 (consistent selection of the delayed reward) to 0.25 (consistent selection of the immediate reward); hence, the higher the *k* is, the more the individual discounts delayed reward.

#### Behavioral Measures of Motor Inhibition

##### Stop-Signal Task

The STOP-IT software was used to assess action stopping (50). Overall, the task consisted of 32 practice trials, followed by three experimental blocks of 96 trials. On each trial, participants were presented with an arrow (go signal); their task was to press the left arrow key of a keyboard with the left index finger when they saw a left arrow, and to press the right arrow key with the right index finger when they saw a right arrow. However, on 25% of the trials (stop trials), the arrow became blue after a variable delay (stop-signal delay; SSD), notifying participants to abort their response. The SSD was initially set at 250 ms, and was continuously adjusted *via* a standard adaptive tracking procedure (i.e. decrease of 50 ms after a successful stop and increase of 50 ms after an unsuccessful stop); this converges on a response rate to a stop trial of ±50%. Importantly, participants were instructed to respond as accurately and as fast as possible (maximal reaction time [RT] set at 1,250 ms), and not to delay their response to wait for the potential onset of a stop-signal. The stop-signal reaction time (SSRT), which corresponds to the latency of the stop process, was estimated with the integration method (with replacement of go omissions, i.e. 0.007% of the trials in the current study), such as recently recommended by Verbruggen et al. (51). In addition to the SSRT, we measured the RTs on Go trials, RTs on unsuccessful stop trials, and the SSDs.

##### Anti-Saccade Task

In this task [adapted from (52)], participants performed three different blocks, all of them involving a similar procedure. Each trial started with the presentation of a fixation cross in the middle of the screen for 1,500 to 3,500 ms, followed by the onset of a target stimulus. This stimulus was an arrow inside a square displayed for 150 ms on the left or the right side of the screen, before being masked by a gray cross-hatching square. The participant's task was to indicate the orientation of the arrow (towards the left, the right, or upwards) by pressing the corresponding key on a keyboard. The first two blocks corresponded to control conditions, whereas the third one was the experimental condition. In the first block (No cue [NC]; 40 trials), the sequence of events for each trial occurred as described above. In the second type of block (Congruent cue [CC]; 40 trials), a visual cue (a black square) was presented for 225 ms between the fixation cross and the target stimulus on the same side as the arrow. Finally, the last block involved trials in which the visual cue was systematically displayed on the side opposite to the target stimulus (Incongruent cue [IncC]; 80 trials). Given that the arrow appeared for only 150 ms, participants had to inhibit the automatic response triggered by the IncC in order to correctly identify the orientation of the arrow. The critical measure was the anti-saccade cost, which was computed for both RTs and percentage of correct responses, by calculating the difference between the average scores obtained in the IncC block and the average scores recorded in the two other NC and CC blocks.

#### Neural Measures of Preparatory Suppression

##### The “Rolling Ball” Task

**Figure 1:**
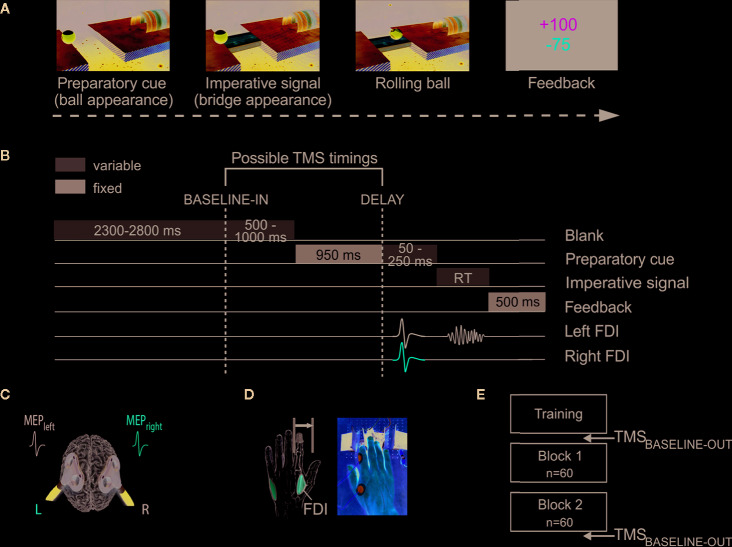
Experimental procedure to measure preparatory suppression. **(A)** Rolling ball task. Subjects performed an instructed-delay choice reaction time task, requiring them to choose between an abduction movement of the left or right index ﬁnger (left in the current example) depending of the position of a preparatory cue (i.e., the ball). They had to withhold their response until the onset of an imperative signal (i.e., the bridge). Once the bridge appeared, they were required to release their response as fast as possible. If they answered correctly, the ball then rolled over the bridge and reached the goal located on the other side. A feedback reflecting how fast and accurate subjects had been concluded each trial. **(B)** Time course of a trial. Each trial started with the preparatory cue (random duration; 1,000–1,200 ms) followed by the imperative signal, which remained visible until the subject responded (maximum duration of 700 ms). The feedback was presented at the end of each trial for 500 ms. Transcranial magnetic stimulation (TMS) was used to elicit motor-evoked potentials (MEPs) in the ﬁrst dorsal interosseous (FDI) of both hands. TMS pulses could occur either during the inter-trial interval (between 2300 and 2800 ms after the blank screen onset; TMS_BASELINE-IN_), or during the delay period (950 ms after the preparatory cue onset; TMS_DELAY_). **(C)** TMS protocol. Two ﬁgure-of-eight coils were placed over the subject's primary motor cortex, eliciting near simultaneous MEPs (1ms delay) in the left and right FDIs. **(D)** Response device. Index ﬁnger responses were recorded using a home-made response device positioned under the left (graphic representation) and right (photographic representation) hands. **(E)** Time course of the experiment. After a training block, subjects performed two blocks of 60 trials. Moreover, MEPs were elicited before and after the two experimental blocks to obtain a measure of corticospinal excitability outside the context of the task (TMS_BASELINE-OUT_).

Participants performed an instructed-delay choice RT task, which was implemented with Matlab 7.5 (Mathworks, Natick, Massachusetts, USA) using the Psychophysics Toolbox extensions (53, 54). It consists in a virtual “rolling ball” game previously used in other studies [ (8, 9, 17, 55); see **Figure 1A**]. In this task, a ball and a goal appear on a computer screen and participants must virtually “shoot the ball into the goal” by performing an abduction movement with the left or right index finger, which requires the activation of the left or right first dorsal interosseous (FDI) muscle, respectively.

The sequence and timing of events are shown in **Figure 1B**. Each trial started with the presentation of a preparatory cue, consisting of a ball and a goal separated by a gap. Participants had to prepare an abduction of the left index finger when the ball was displayed on the left side of the screen, and an abduction of the right index finger when it appeared on the right. Subjects were explicitly told to withhold their prepared response until the onset of an imperative signal (i.e., the bridge), which appeared 1,000 to 1,200 ms later. We purposely varied the duration of the delay period to decrease the subjects' tendency to respond prematurely (i.e., before the imperative signal). For the same reason, each block involved a few trials in which the bridge did not appear (i.e., catch trials—4 per block). In these trials, subjects were required not to respond and were penalized if they did so. Once the bridge was on the screen, subjects had to respond as fast as possible to make the ball roll over it, within a maximum time of 700 ms. The imperative screen disappeared once a response was detected (or after 700 ms) and a feedback was presented for 500 ms. Following a correct response, the feedback consisted of a positive score depicted in green, which ranged from 1 to 100 and was inversely proportional to the trial's RT $\left(\text{Score}=\frac{100*\left(0,8*250\right)}{\left(0,8*250\right)+{\left(\frac{RT-\left(0,8*250\right)}{10}\right)}^{2,4}}\right)$. By contrast, incorrect responses (i.e., responses provided prematurely, that is before the onset of the imperative, responses provided too late, that is more than 700 ms after the imperative onset, or responses that were provided with the incorrect finger) were penalized by a negative score (−75) displayed in red. Note that when subjects succeeded not to respond on a catch trial, they received +75 points. Finally, each trial ended up with a blank screen, lasting between 2,800 and 3,800 ms (inter-trial interval).

##### TMS Protocol

TMS was always delivered using a double-coil TMS method recently developed in our laboratory (17, 56–58), where both M1 are stimulated with a 1 ms inter-pulse interval, eliciting MEPs in both hands at a near simultaneous time (**Figure 1C**). Adding an interval between both pulses allows to avoid direct electromagnetic interference between the two coils, while keeping it short prevents transcallosal interactions to occur between motor areas. The MEPs obtained using this double-coil approach are comparable to those elicited using single-coil TMS, regardless of the pulse order or the intensity of stimulation (17, 56); here, the first pulse was systematically applied over right M1. Both pulses were delivered through small figure-of-eight coils (wing internal diameter 35 mm), each connected to a stimulator delivering monophasic pulses. The coils were placed tangentially on the scalp with the handle pointing backward and laterally at 45° angle away from the midline, approximatively perpendicular to the central sulcus. For each M1, the optimal coil position for eliciting MEPs in the contralateral FDI was identified and marked on a head cap placed on the participant's scalp to provide a reference mark throughout the experiment (8, 18, 59).

The resting motor threshold (rMT) was determined at the hotspot for each M1 as the minimal TMS intensity required to evoke MEPs of 50 μV peak-to-peak in the relaxed FDI muscle in 5 out of 10 consecutive stimulations. Across control subjects, the rMTs corresponded to 41.92 ± 2.56% and 41.46 ± 2.65% of the maximum stimulator output for the left and right M1, respectively. In GDPs, the rMTs equaled 41.77 ± 2.58% and 41.53 ± 2.79% in the corresponding conditions. The intensity of TMS used throughout the experiment was always set at 115% of the individual rMT for each hemisphere.

##### Experimental Design

Participants sat in front of the computer screen with forearms resting in a semi-flexed position and hands placed palms down on a home-made response device developed in our laboratory to detect any horizontal movement of the index fingers (**Figure 1D**). This setup provides us with a very precise measure of the RT (precision = 1 ms) and allows us to control the initial index finger position at the beginning of each trial [for more details regarding this device, please refer to (60)].

As illustrated in **Figure 1E**, the testing always began with a training block in order to familiarize the subjects with the task, followed by two experimental blocks of 60 trials. During those blocks, TMS pulses were delivered at one of two possible timings (**Figure 1B**). To establish a baseline measure of corticospinal excitability (CSE), TMS pulses fell during the inter-trial interval, between 2,300 and 2,800 ms after the blank screen onset (i.e., 500 to 1,000 ms before the onset of the preparatory cue), eliciting MEPs at rest but in the context of the task (TMS_BASELINE-IN_; 18 MEPs per block). In other trials, TMS pulses were delivered 950 ms after the onset of the preparatory cue, when subjects were withholding their response (TMS_DELAY_; 18 MEPs per responding side and per block). The remaining trials (six per block) did not include any TMS pulse, preventing participants from anticipating TMS pulses at TMS_DELAY_ when it had not occurred at TMS_BALSINE-IN_. Finally, 18 TMS pulses were applied before and after the two experimental blocks to obtain a baseline measure of CSE at rest outside the context of the task (TMS_BASELINE-OUT_).

##### Electromyography (EMG) Recording

EMG activity was recorded from surface electrodes (Ambu Blue Sensor NF-50-K Neuroline, Medicotest, Oelstykke, Denmark) placed over the FDI muscle of the left and right hands. EMG data were collected for 3,200 ms on each trial, starting 200 ms before the TMS pulse. The raw EMG signals were amplified (gain, 1 K), bandpass filtered online (10–500 Hz, NeuroLog; Digitimer) and digitized at 2,000 Hz for offline analysis. The latter consisted in extracting the peak-to-peak amplitude of MEPs recorded in both FDIs. In order to prevent contamination of MEP measurements by significant fluctuations in background EMG, trials with EMG activity (root mean square computed in the 200 ms windows preceding the TMS pulse) exceeding 2.5 standard deviations (SD) around the mean were discarded from the following analyses (8, 17). The remaining MEPs were classified according to the experimental condition within which they had been elicited. Trials in which subjects made an error were also removed from the data set; the task was so easy that these trials remained rare and errors were not analyzed. For each condition, we excluded trials with peak-to-peak MEP amplitudes exceeding 2.5 SD around the mean. Following data cleaning, a mean of 30.7 ± 3.5 trials per condition were left.

### Statistical Analyses

#### Self-Reported Measures

Demographic variables and current clinical status were compared in HCs and GDPs using independent sample t-tests. Trait impulsivity was analyzed by conducting two separate multivariate analyses of variance (MANOVAs) on scores reported at the different subscales of the BIS-11 and the UPPS questionnaire, with GROUP (HCs, GDPs) as the between-subject factor. To analyze choice impulsivity, a two-way ANOVA was computed on *k*-values obtained at the MCQ, with MAGNITUDE (small, medium, large) as the within-subject factor and GROUP (HCs, GDPs) as the between-subject factor. Importantly, as several works reported that using the natural log (nlog) transformation allows to approximately normalize the distribution of *k*-values (61, 62), analyses were performed on *nlog k-*values.

#### Behavioral Measures of Motor Inhibition

To compare motor inhibition in both groups, independent sample t-tests were performed on the critical measures specific to each task, i.e. the SSRT and the anti-saccade cost. In addition, RTs on Go trials, RTs on unsuccessful stop trials and SSDs were compared using Welch's t-tests, because of unequal variances in HCs and GDPs.

#### Neural Measures of Preparatory Suppression

First, we focused on CSE at rest, by considering MEPs probed outside the blocks (TMS_BASELINE-OUT_) and those recorded within the blocks (TMS_BASELINE-IN_). The raw amplitude of those MEPs (mV) was analyzed using a three-way ANOVA, with MEP-SIDE (Left, Right) and TMS-TIMING (TMS_BASELINE-OUT_, TMS_BASELINE-IN_) as within-subject factors and GROUP (HCs, GDPs) as the between-subject factor. Then, we considered MEPs at TMS_DELAY_; those MEPs were expressed in percentage of MEPs elicited at TMS_BASELINE-IN_. To assess the presence of preparatory suppression in each sub-condition, one-sample t-tests (Bonferroni-corrected) were carried out to compare these values to 100 (i.e. to TMS_BASELINE-IN_). Furthermore, the strength of preparatory suppression was compared between both groups by performing a three-way ANOVA, using MEP-SIDE (Left, Right) and CONDITION (Selected, Non-Selected) as the within-subject factors and GROUP (HCs, GDPs) as the between-subject factor. Finally, to analyze behavior during the rolling ball task, a three-way ANOVA was computed on RTs, with RESPONDING-SIDE (Left, Right) and TMS-TIMING (TMS_BASELINE-IN_, TMS_DELAY_) as within-subject factors and GROUP (HCs, GDPs) as the between-subject factor.

#### Exploratory Analyses on the Relationships With Gambling Severity

In order to assess the potential link between the total number of DSM-V criteria and psychopathological variables as well as our different measures of inhibition in GDPs, partial Pearson's correlations were performed in these subjects, using the factor AGE as a covariate.

The Fisher's Least Significant Difference (LSD) method was used to run post-hoc comparisons. All of the data are expressed as mean ± SE and the statistical significance was set at p < 0.05. Analyses were carried out using Statistica 10 (StatSoft, Cracow, Poland).

## Results

### Demographics and Current Clinical Status

As illustrated in **Table 1**, analyses confirmed that both groups were matched for age (t24 = −0.21, p = 0.67) and education level (t24 = 1.92; p = 0.07). In addition, they did not significantly differ for state anxiety (t24 = −1.29; p = 0.74), trait anxiety (t24 = 0.97; p = 0.45), and depression (BDI, t24 = −1.67; p = 0.11). Finally, the AUDIT score was not significantly different between HCs and GPDs (t24 = 1.71; p = 0.10).

**Table 1:** Demographic and psychopathological measures for healthy controls (HCs) and gambling disorder patients (GDPs) (Mean [SE]).

	HCs (n = 13)	GDPs (n = 13)
Age^NS^	27.8 *(2.4)*	28.6 *(2.7)*
Education level^1,NS^	15.5 *(0.48)*	14.1 *(0.6)*
Trait anxiety^NS^	35.3 *(2.9)*	40.5 *(2.7)*
State anxiety^NS^	43.3 *(3.2)*	43.5 *(2.6)*
BDI^NS^	3.6 *(1.0)*	6.8 *(1.6)*
AUDIT^NS^	6.1 *(0.7)*	4.2 *(0.9)*
Tobacco (n smokers)	0	4
SOGS	0	9.1 *(0.6)*
DSM-V criteria	0	5.8 *(0.4)*

1 The education level reflects the number of years of education completed since starting primary school. BDI, Beck Depression Inventory; AUDIT, Alcohol Use Disorders Identification Test; SOGS, South Oaks Gambling Screen; DSM, Diagnostic and Statistical Manual; NS, non-significant.

### Trait Impulsivity

The MANOVA performed on scores at the BIS-11 questionnaire showed a significant main effect of the factor GROUP (λ_3,22_ = 0.49; *p* < 0.01). As shown in **Table 2**, univariate results obtained for each subscale reveal that the significant difference between both groups was due to higher scores on the non-planning impulsiveness subscale in GDPs relative to HCs (F_1,24_ = 21.21; *p* < 0.001). Moreover, scores on the motor subscale also tended to be higher in GDPs, even if it did not reach significance (F_1,24_ = 3.31; *p* = 0.08).

**Table 2:** Trait impulsivity measures for healthy controls (HCs) and gambling disorder patients (GDPs) (Mean [SE]).

	HCs (n = 13)	GDPs (n = 13)
BIS-11**		
Attentional	17.1 *(1.2)*	17.3 *(0.9)*
Motor	20.5 *(1.0)*	23.3 *(1.2)*
Non-planning***	21.2 *(1.0)*	27.2 *(0.8)*
**UPPS Scale^NS^**		
Urgency	26.6 *(1.5)*	29.7 *(1.8)*
Lack of premeditation	20.1 *(1.1)*	23.9 *(0.9)*
Lack of perseverance	19.1 *(1.5)*	21.4 *(1.4)*
Sensation seeking	35.2 *(1.8)*	35.3 *(2.5)*

NS, non-significant. **p < 0.01 and ***p < 0.001.

Surprisingly, the main effect of GROUP was not significant for the UPPS scale (λ34,21 = 0.70; p = 0.10). Nonetheless, it is interesting to note that univariate analyses still reveal a significant difference between both groups for the lack of premeditation subscale (F1,24 = 6.79; p < 0.05), consistent with the results regarding the BIS-11 questionnaire

### Choice Impulsivity

**Figure 2:**
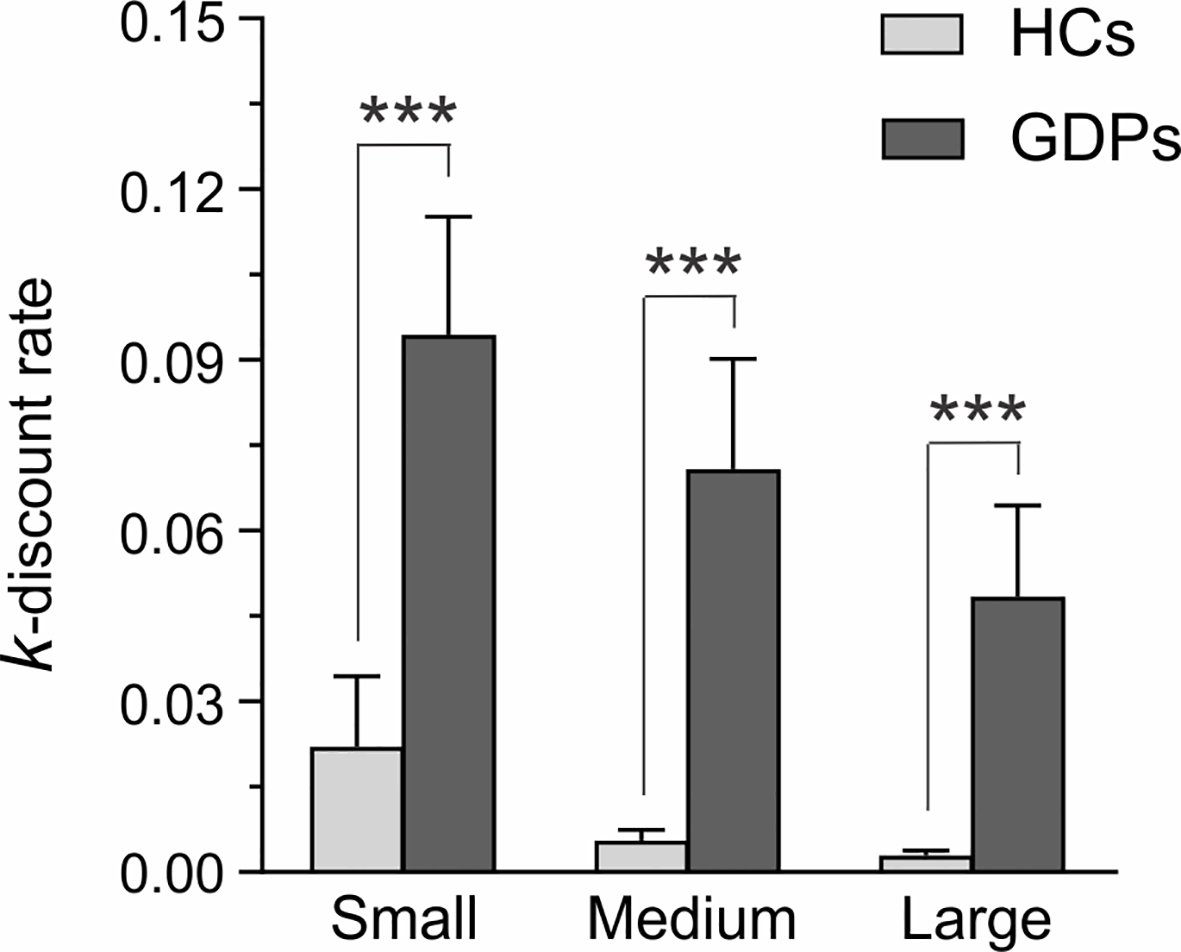
Self-reported measures for choice impulsivity. K-values estimated from the Monetary Choice Questionnaire (MCQ) are shown for HCs (light gray) and GDPs (dark gray) when the delayed reward was small, medium, or large. Higher k-values reflect higher discounting rates. Please note that statistical analyses were performed on nlog k-values, although this figure depicts the raw data. Those results highlight the higher discount rate in GDPs relative to HCs, regardless of the magnitude of the delayed reward. ***p < 0.001: significantly different.

The ANOVA performed on the *nlog k*-values of HCs and GDPs revealed a significant main effect of GROUP (F_1,24_ = 22.17; *p* < 0.001). Hence, and as evident on **Figure 2**, GDPs discounted reward at a significantly higher rate than HCs did. Furthermore, the main effect of MAGNITUDE was significant (F_2,48_ = 15.03; *p* < 0.001), which reflected a decrease in discounting rates as the amount of the delayed reward increased. Interestingly, the interaction between both factors was not significant (F2,48 = 0.13; p = 0.73), indicating that GDPs had higher discounting rates relative to controls regardless of the reward amount. Accordingly, further analyses performed on the mean discounting rate estimated for the whole questionnaire (i.e. 0.006 ± 0.002 and 0.056 ± 0.018 for HCs and GDPS, respectively) revealed a significant difference between both groups (t24 = −4.47; p < 0.001).

### Behavioral Measures of Motor Inhibition

**Figure 3:**
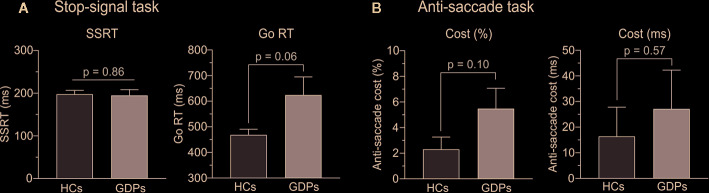
Behavioral measures of motor inhibition. **(A)** Stop-signal reaction time (SSRT) and reaction time (RT) on go trials during the stop-signal task in HCs (light gray) and GDPs (dark gray). **(B)** Anti-saccade cost, defined as the difference between the scores (% of errors and RTs) in incongruent and control trials, in HCs and GDPs (same color code).

In the stop-signal task, two GDPs did not properly follow the instructions—i.e. probability of responding on a stop trial higher than 0.75—and were consequently excluded from the subsequent analyses, as recommended (51). In the remaining subjects, the mean response rate on stop trials equaled 49.17 and 47.76% in HCs and GDPs, respectively, indicating that the tracking procedure was successful, which should allow a valid interpretation of the SSRT. As shown on the left panel of **Figure 3A**, the t-test performed on this index revealed no significant difference between both groups (t22 = 0.18; p = 0.86), suggesting similar abilities to abort an ongoing action in HCs and GDPs. However, GDPs seemed to be slower than controls when performing the task, such as indicated by longer RTs on Go trials (t22 = −2.23; p = 0.06; see right panel of **Figure 3A**). This overall slowness was also reflected in measures of the SSD (243.7 ± 23.9 and 396.3 ± 75.6 ms in HCs and GDPs, respectively; t22 = −1.92; p = 0.07) and of RTs on unsuccessful stop trials (393.9 ± 10.9 and 523.5 ± 60.1 ms in HCs and GDPs, respectively; t22 = −2.12; p = 0.06).

Regarding the anti-saccade task, even though the figures suggest a larger cost in GDPs than in controls, our analyses did not reveal any significant difference between both groups, neither for the percentage of correct responses (t24 = −1.71; p = 0.10; left panel of **Figure 3B**) nor for the RTs (t24 = −0.57; p = 0.57; right panel of **Figure 3B**). Hence, GDPs did not significantly display more difficulties than HCs to inhibit the initial reflexive saccade towards the incongruent visual cue.

### Neural Measures of Preparatory Suppression

#### MEP Measurements

**Figure 4:**
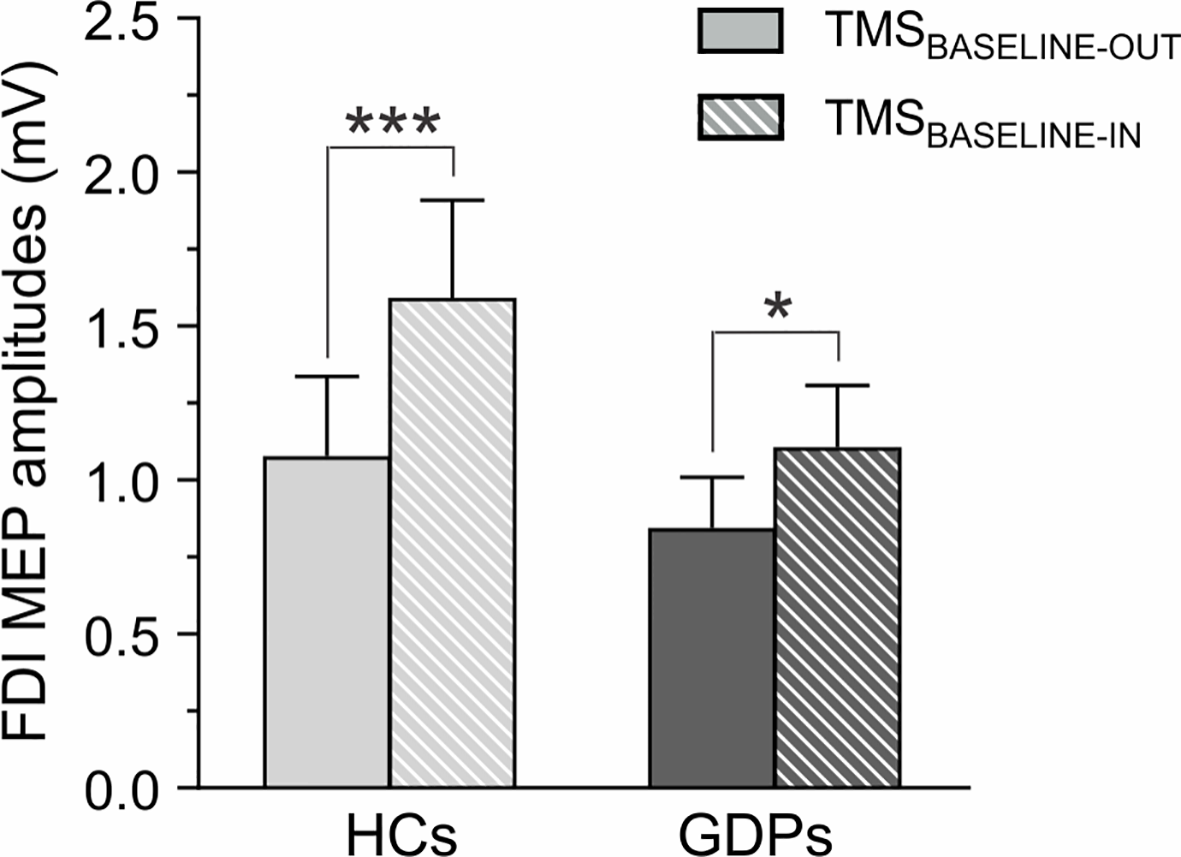
Measures of corticospinal excitability (CSE) at rest. Raw amplitude of motor-evoked potentials (MEPs, in mV) recorded in the FDI at rest, either outside (TMS_BASELINE-OUT_; open bars) or within (TMS_BASELINE-IN_; dashed bars) the blocks. MEPs are shown for HCs (light gray) and GDPs (dark gray). Such as revealed by the significant GROUP X TMS-TIMING interaction, the increase in CSE during the task was larger in HCs than in GDPs. *p < 0.05 and ***p < 0.001: significantly different.

First, we considered MEPs acquired at rest, either outside or within the blocks (TMS_BASELINE-OUT_ and TMS_BASELINE-IN_). Overall, the amplitude of MEPs probed at TMS_BASELINE-OUT_ was 1.06 ± 0.26 mV and 0.84 ± 0.16 mV in HCs and GDPs, respectively. When elicited at TMS_BASELINE-IN_, MEPs equaled 1.64 ± 0.34 mV and 1.06 ± 0.21 mV in the corresponding groups. In line with previous studies (8, 17, 55), MEPs were globally larger at TMS_BASELINE-IN_ relative to TMS_BASELINE-OUT_ (F1,24 = 39.14; p < 0.001), reflecting an increase in the level of CSE in the context of the task. However, consistent with the significant GROUP X TMS-TIMING interaction (F1,24 = 8.07; p < 0.01) and as shown on **Figure 4**, this increase was more pronounced in controls (p < 0.001) than in GDPs (p < 0.05).

**Figure 5:**
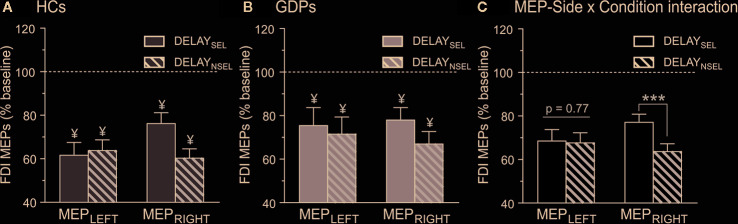
Neural measures of preparatory suppression. Amplitude of motor-evoked potentials (MEPs) recorded at TMS_DELAY_, expressed in percentage of MEPs elicited at TMS_BASELINE-IN_, shown for the left (MEP_LEFT_) and the right (MEP_RIGHT_) FDI, which was either selected (DELAY_SEL_; open bars) or non-selected (DELAY_NSEL_; dashed bars) for the forthcoming response in HCs **(A)** and GDPs **(B)**. ¥ = significantly different from MEPs probed at TMS_BASELINE-IN_. **(C)** MEP-Side x Condition interaction. MEPs were larger in the selected relative to the non-selected condition only in the right FDI, regardless of the group. ***p < 0.001: significantly different.

Then, we evaluated the amplitude of MEPs elicited during action preparation (expressed in percentage of MEPs at TMS_BASELINE-IN_). As evident on **Figure 5A**, MEPs probed in control subjects were drastically decreased at TMS_DELAY_ when compared to TMS_BASELINE-IN_. This effect occurred for MEPs in both the left and the right FDIs, regardless of whether the muscle was selected or non-selected for the forthcoming response (all t12 < −4.92 and all p < 0.0125). Hence, HCs displayed a strong MEP suppression during action preparation, such as extensively shown in many other works (15, 59, 63). Interestingly, MEPs at TMS_DELAY_ were also significantly suppressed in all conditions in GDPs (all t12 < −3.02 and all p < 0.0125), indicating that those subjects displayed preparatory suppression as well (**Figure 5B**). Moreover, as revealed by the ANOVA computed on these data, the strength of this MEP suppression was comparable in both groups (F1,24 = 1.58; p = 0.22).

Besides, analyses showed a significant main effect of CONDITION (F1,24 = 4.46; p < 0.05), due to a weaker MEP suppression in an effector that was selected for the forthcoming response relative to when the effector was non-selected. However, and as shown on **Figure 5C**, this effect depended on the hand within which the MEPs were elicited (CONDITION x MEP-SIDE interaction F1,24 = 7.64; p < 0.05), as it concerned the right (p < 0.001) but not the left (p = 0.78) hand, regardless of the group (GROUP X MEP-SIDE X CONDITION interaction, F1,24 = 1.44; p = 0.24).

#### Behavior

**Figure 6:**
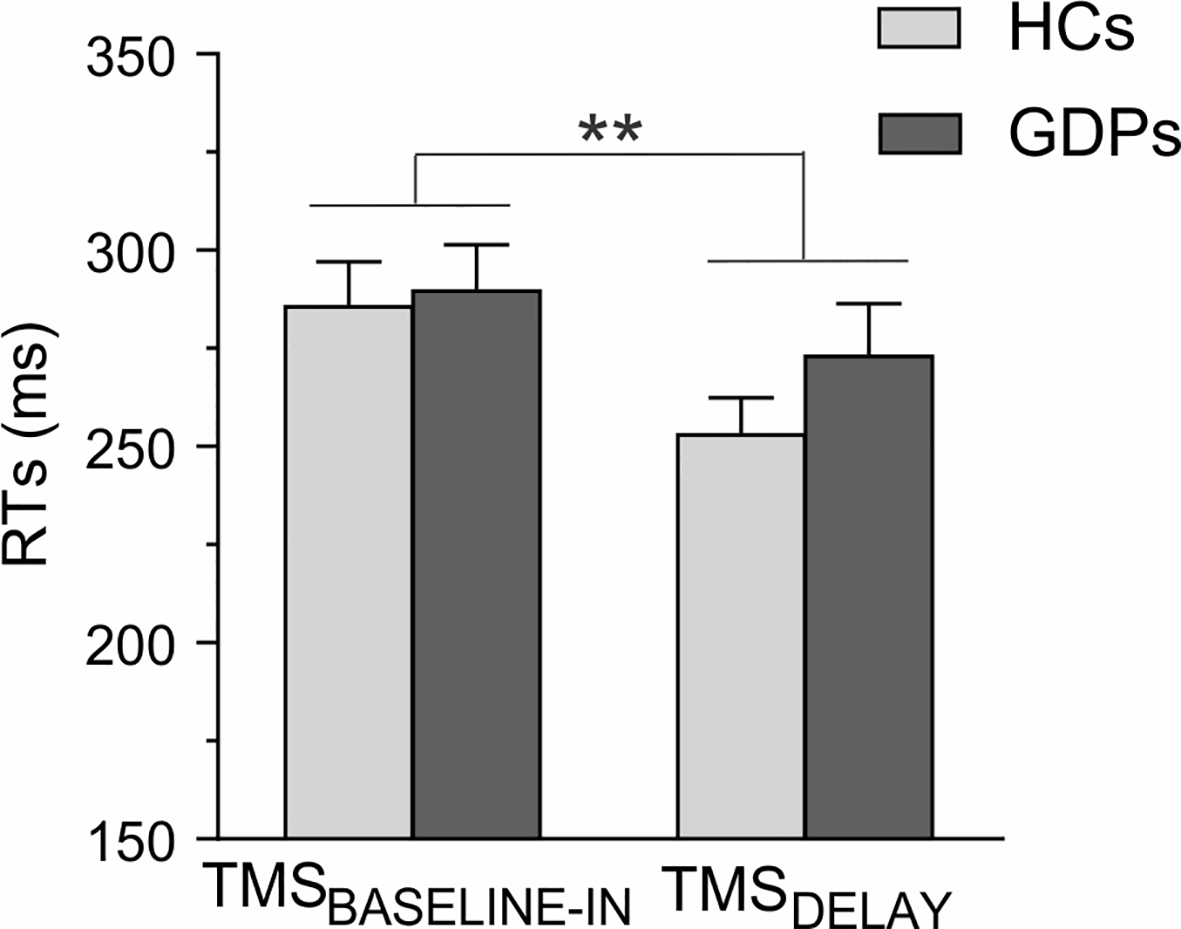
Reaction times (RTs) during the rolling ball task. The RTs are shown for trials in which the TMS pulses were applied either at baseline (TMS_BASELINE-IN_) or during action preparation (TMS_DELAY_) for HCs (light gray) and GDPs (dark gray). Data from responses performed with both hands are pooled together, as the main effect of RESPONDING-SIDE was not significant (F_1,24_ = 0.02; p = 0.89). Please note the shortening of RTs at TMS_DELAY_. **p < 0.01: significantly different.

The RTs measured during the rolling ball task are shown in **Figure 6**. The ANOVA computed on these data revealed a main effect of TMS-TIMING (F1,24 = 13.05; p < 0.01): RTs were faster with TMS_DELAY_ than with TMS_BASELINE-IN_, consistent with many previous reports showing that a TMS pulse applied close to the imperative signal can speed up the release of a motor response (11, 17, 59). By contrast, RTs were comparable in both groups (F1,24 = 0.71; p = 0.41), regardless of the TMS timing (GROUP X TMS-TIMING interaction; F1,24 = 1.38; p = 0.25), indicating that HCs and GDPs performed equally in the task.

### Exploratory Analyses on the Relationships With Gambling Severity

**Figure 7:**
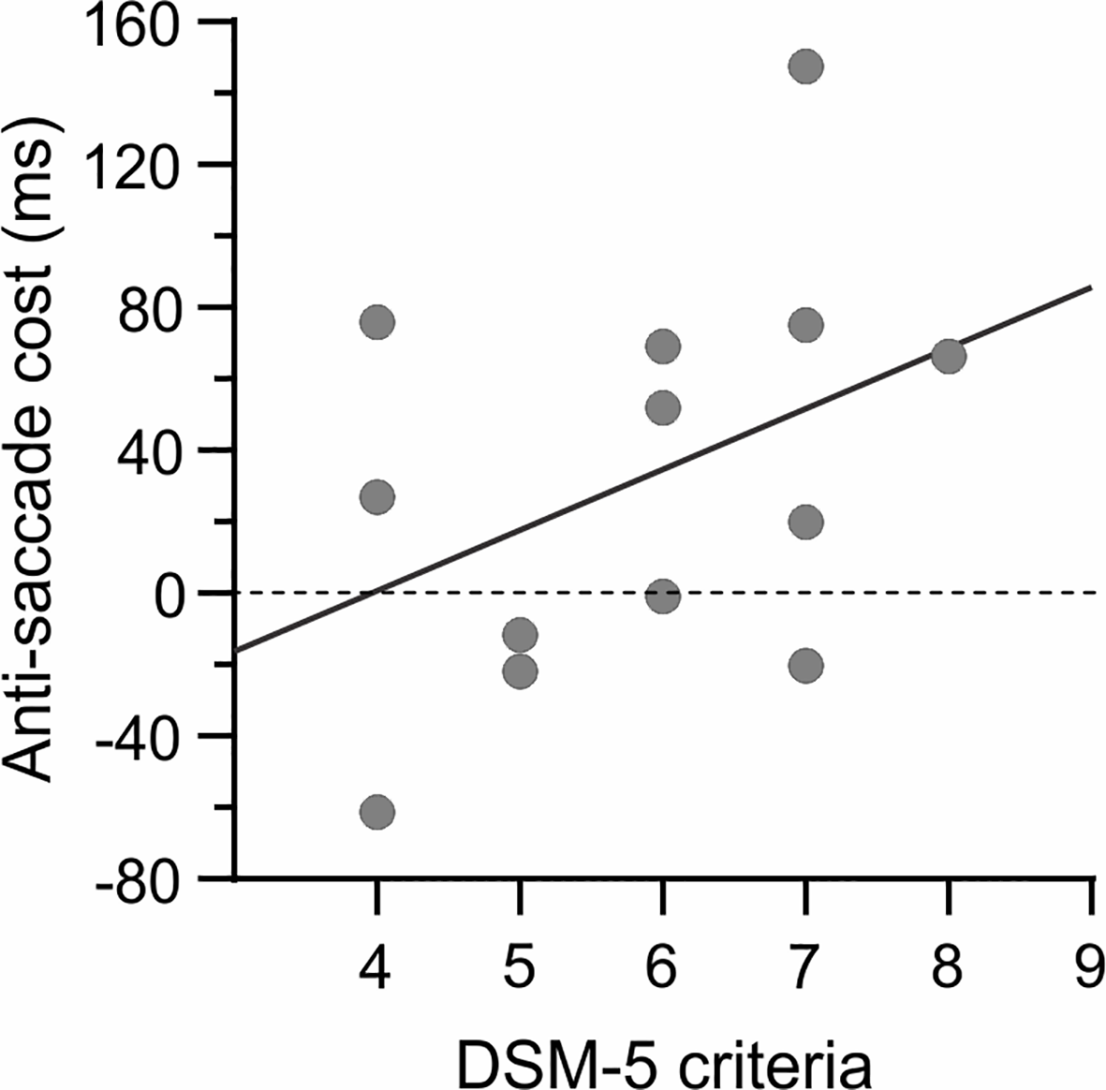
Relationship with gambling severity. A positive correlation (r = 0.65) was observed between the number of DSM-V criteria and the anti-saccade cost (in terms of RT), suggesting lower motor inhibition abilities in more severe GDs. However, note that the correlation did not survive Bonferroni correction.

Regarding the psychopathological variables, the total number of DSM-V criteria significantly correlated with the scores for state anxiety (r = 0.84; p < 0.001) and BDI (r = 0.62; p < 0.05), which indicates that more severe GDs were associated with higher anxiety and depression. Moreover, we observed a significant positive correlation between the DSM-V criteria and the anti-saccade cost in terms of RTs (r = 0.65; p < 0.05; **Figure 7**), suggesting lower motor inhibition abilities in more severe GDs. Nonetheless, note that only the relationship between gambling severity and state anxiety remained after correction for multiple comparisons. None of the other correlations was significant (all −0.37 < r < 0.55 and p > 0.06).

## Discussion

The fifth edition of the DSM recently reclassified pathological gambling from the “Impulse Control Disorder” category to the newly established “Substance-related and Addictive Disorders” section (39). Such a decision implies that this substance-free gambling addiction shares many features with addictions to substances (64–66). In this context, extensive work is being dedicated to understanding these similarities and to identifying vulnerability markers that may be common to all addictive disorders, with one particularly promising candidate being a lack of inhibitory control. In the present study, we addressed this question by assessing preparatory suppression, a specific facet of inhibitory control, in a group of GDPs and in matched HCs. Contrary to our hypothesis, GDPs did not lack any preparatory suppression, though they had some deficits in other aspects of inhibitory control, such as a steeper delay discounting rate and a higher trait impulsivity than HCs.

In control subjects, MEPs elicited during the rolling ball task were globally smaller at TMS_DELAY_ than at TMS_BASELINE-IN_, consistent with the literature reporting a lower corticospinal excitability during action preparation (5). Also, in line with some previous studies [(57, 59); but see also (17)], this preparatory suppression was less prominent in the dominant hand, especially when it was selected for the subsequent movement, possibly reflecting a higher readiness of effectors from the dominant hand to initiate actions (57, 58). Interestingly, the same pattern of results was found in GDPs, suggesting that those subjects did not suffer from a deficit in preparatory suppression. In fact, the only group difference that we found during the rolling ball task concerns MEPs elicited during the inter-trial interval, at TMS_BASELINE-IN_. In both groups, these MEPs were larger than those elicited outside the blocks, at TMS_BASELINE-OUT_, in agreement with previous studies (17, 55, 63). Yet, this increase was more pronounced in HCs than in GDPs. This difference might be the result of dissimilarities at the level of attention, vigilance, or arousal (2, 67). As such, one possibility is that GDPs are less motivated by a task in which the sole reward consisted in a feedback score, leading to a blunted resting excitability during the blocks. Accordingly, fMRI studies have reported a diminished sensitivity towards small or non-monetary rewards in gamblers relative to control subjects (68, 69). Nevertheless, this plausible reduced vigilance in GDPs did not impact the level of preparatory suppression or even the performance in the rolling ball task.

While we did not observe an alteration of preparatory suppression in GDPs, there were some differences between both groups regarding some aspects of impulsivity, substantiating the presence of a deficit as expected in these patients. Although multiple theoretical models have been put forward (70–72), impulsivity is commonly divided into two primary components, called *choice impulsivity* and *rapid response impulsivity* (73, 74). In line with the relative independence of those two types of impulsivity, GDPs were affected differently depending on the assessed component. Our findings at the MCQ revealed that GDPs had significantly steeper discounting rates than HCs, highlighting a higher choice impulsivity in this group of patients, as shown previously (35, 75, 76). This predisposition to prefer smaller-sooner rewards over larger-delayed ones was also supported by our measures of trait impulsivity. Indeed, GDPs obtained higher scores than controls on the “non-planning” and “lack of premeditation” subscales of the BIS-11 questionnaire and the UPPS Impulsive Behavior Scale, respectively, implying that those individuals are more oriented to the present rather than the future and tend to act without consideration of the consequences. In this line, an association between scores on both subscales and discounting rates has been previously reported (61, 76, 77). Altogether, this pattern of results is likely to contribute to the tendency of GDPs to favor immediate bets, despite the negative consequences of their gambling behavior.

By contrast, GDPs and HCs did not seem to differ at the level of rapid response impulsivity. This lack of group effect concerned not only behavioral motor inhibition, such as assessed by the stop-signal and the anti-saccade tasks, but also impulsivity trait, as scores at the motor subscale of the BIS-11 questionnaire were not significantly different between both groups. This result contrasts with recent meta-analyses suggesting higher rapid response impulsivity in subjects suffering from pathological gambling (30, 78), although some studies failed to identify a deficit (79, 80). Nonetheless, the present results need to be put into perspective in several ways. First, while the SSRT was similar in HCs and GDPs, it is noteworthy that other task variables estimated in the stop-signal task, such as the RTs on Go trials and unsuccessful stop trials, as well as the SSDs, indicated a general slowness to respond in GDPs. Hence, it might be that GDPs strategically slowed down in anticipation of the stop-signals to compensate for a potential inhibitory deficit, even though blocks without stop-signals would have been required to ascertain this hypothesis. Besides, the slowness reported in GDPs might have prevented us from highlighting a lack of response inhibition. Indeed, while the tracking procedure was efficient, the SSD had to drastically increase to adapt to the slower responses of GDPs. Consequently, the number of trials was probably insufficient in the present study to allow the SSD to reach a relatively steady value at which the SSRT can be computed reliably. Hence, future studies should use initially longer SSDs to observe a potential deficit in GDPs (35). Moreover, despite the overall lack of group effect in the anti-saccade task, we found an interesting positive correlation between the number of DSM-V criteria and the anti-saccade cost, which was quite variable in GDPs. In fact, and in line with previous works (35, 36, 81), this association indicates that a deficit in rapid response impulsivity only concerned the most severe forms of pathological gambling, contrary to choice impulsivity, which was higher regardless of gambling severity. Finally, it is worth noting that GDPs tended to score higher at the motor subscale of the BIS-11 questionnaire, and that the lack of significant difference is likely to result from our small sample size.

The current findings in GDPs contrast considerably with our prior observations in alcohol-dependent patients (ADPs), which revealed a major lack of preparatory suppression, especially in the patients who relapsed during the year following the testing (9). As GDPs are preserved from the neurotoxic influence of drugs of abuse, one straightforward interpretation of the discrepancy between both studies is that the lack of preparatory suppression observed in ADPs arises as a consequence of chronic alcohol consumption. Accordingly, it has been shown that the lateral prefrontal cortex—i.e., the region of the alcoholic brain in which the decrease in gray matter volume is the most significant (82)—generates at least part of the preparatory suppression effect (11, 83). Hence, it is plausible that this prefrontal source of preparatory suppression is specifically reduced in ADPs after years of alcohol abuse. Future studies, combing structural magnetic resonance imaging to quantify the brain damage and TMS to assess preparatory suppression in ADPs, are required to conclude on this point. Moreover, it would be interesting to investigate whether the lack of preparatory suppression extends to other substance use disorders. Yet, it is noteworthy that structural and functional prefrontal alterations still exist in GDPs (84–88), even in the absence of any substance use disorder comorbidity (89). In particular, an increasing literature has led to consider the neuromodulation of the dorsolateral prefrontal cortex, using high-frequency repetitive TMS, as a potential treatment for GD (66), with encouraging results in terms of craving reduction (90–92), but not regarding inhibitory control (93). That being said, the absence of deficit in preparatory suppression reported in GDPs makes it an unlikely common vulnerability marker of whether one is going to develop an addiction or not.

The present finding that preparatory suppression was comparable in GDPs and HCs should be interpreted with caution and requires further evidences as the current study suffer from several limitations. First, GDPs represent a highly heterogeneous group, characterized by different impairments depending on the form of gambling in which they engage (32, 94). Although impulsivity is an important ethiological factor for GD, the recognized pathway model of Blaszczynski and Nower (95) posits that it represents only one of the three routes than can lead to pathological gambling. Hence, it is likely that our results reflect the average of different GD profiles, and that our findings would have been more similar to those observed in ADPs if we had only included GDPs from the impulsivity pathway. Second, the power of our study is rather low given our small sample sizes. Yet, this is the best we could do in view of the real challenge of recruiting patients suffering of GD *only*. Indeed, many GDPs show high extent of comorbidity with alcohol and substance use disorders (96), preventing them from participating in experiments aiming at assessing the neuropsychological profile exclusively associated with GD. Finally, our sample was entirely male. Although this is consistent with gender biases in the GD population (30, 76, 94), this limits the generalizability of our findings to females.

In summary, although we found some alterations in several aspects of inhibitory control in the sample of GDPs tested in the current study, preparatory suppression was not deficient in these patients. This finding contrasts with prior observations reported in subjects suffering from an alcohol use disorder, suggesting that a lack of preparatory suppression does not represent a common feature shared by behavioral and substance-related addictions. Critically, future studies would gain from taking into account the large heterogeneity in GDP profiles and possibly focus on patients that are part of the impulsivity pathway. Moreover, extending investigations of preparatory suppression to other “Substance-related and Addictive Disorders” should further our understanding of inhibitory control as a vulnerability marker underlying these conditions.

## Data Availability Statement

The datasets generated for this study are available on request to the corresponding author.

## Ethics Statement

This study was reviewed and approved by Biomedical Ethic Committee of the Saint-Luc University Hospital, Université catholique de Louvain. The patients/participants provided their written informed consent to participate in this study.

## Author Contributions

CQ, JG, and JD: designed the experiment. CQ and JG: performed the experiments. CQ and JG: analyzed data. CQ and JD: wrote the article.

## Funding

This work was supported by grants from the Belgian National Funds for Scientific Research (FRS-FNRS) and the “Fondation Médicale Reine Elisabeth” (FMRE). CQ was a post-doctoral fellow supported by the FNRS. JG was a graduate student supported by a Fund for Research Training in Industry and Agriculture (FRIA). JD is a Professor at the Institute of Neuroscience of the Université catholique de Louvain.

## Conflict of Interest

The authors declare that the research was conducted in the absence of any commercial or financial relationships that could be construed as a potential conflict of interest.
